# Enhancing Motor Function and Quality of Life Combining Advanced Robotics and Biomechatronics in an Adult with Dystonic Spastic Tetraparesis: A Case Report

**DOI:** 10.3390/biomimetics10020113

**Published:** 2025-02-14

**Authors:** Elisabetta Leogrande, Sara Piccoli, Francesco Dell’Olio, Nicola Smania, Stefano Mazzoleni, Marialuisa Gandolfi

**Affiliations:** 1 Micro Nano Sensor Group, Politecnico di Bari, 70126 Bari, Italy; e.leogrande1@phd.poliba.it (E.L.); francesco.dellolio@poliba.it (F.D.); 2 Department of Neuroscience, Biomedicine and Movement Sciences (DNBM), Università di Verona, 37134 Verona, Italy; sarapiccoli86@gmail.com (S.P.); nicola.smania@univr.it (N.S.); 3 Neuromotor and Cognitive Rehabilitation Research Centre (CRRNC), 37134 Verona, Italy; 4 Department of Electrical and Information Engineering, Politecnico di Bari, 70126 Bari, Italy; stefano.mazzoleni@poliba.it; 5 IMT School for Advanced Studies Lucca, 55100 Lucca, Italy; 6 The BioRobotics Institute, Scuola Superiore Sant’Anna, 56025 Pontedera, Italy

**Keywords:** neuromodulation, rehabilitation, robotics, medical treatments

## Abstract

This case report explores the innovative integration of robotic and biomechatronic technologies, including the Motore and Ultra+ devices and neuro-suits, in a 10-session rehabilitation program for a young adult with dystonic spastic tetraparesis. Notable improvements were observed in upper limb motor function, coordination, and quality of life as measured by an increase of 18 pints on the Fugl-Meyer scale and a 25% improvement in the Bartle Index. Range of motion measurements showed consistent improvements, with task execution times improving by 10 s. These findings suggest the potential of combining wearable, robotic, and biomechatronic systems to enhance neurorehabilitation. Further refinement of these technologies might support clinicians in maximizing their integration in therapeutics, despite technical issues like synchronization issues that must be overcome.

## 1. Introduction

Neurological rehabilitation is a fundamental pillar of rehabilitative medicine, addressing motor, cognitive, and sensory impairments caused by central or peripheral nervous system damage within a multidisciplinary framework. Traditional approaches have progressively been enhanced by robotic and biomechatronic technologies, which offer precise, adaptable, and task-oriented interventions tailored to individual needs [1,2,3]. These systems enable immersive and interactive rehabilitation, providing real-time feedback and promoting motor learning through intensive repetition and task-specific exercises, particularly with the introduction of robotic systems equipped with adaptive control [1]. Of note, the task-oriented approach mirrors real-world activities, enhancing the relevance of exercises.

This bioinspired principle draws on how the human nervous system learns and adapts to repeated, purposeful actions, similar to natural motor learning processes [4].

Such innovations have been pivotal in treating the most disabling neurological conditions, such as stroke and neurodegenerative diseases, and, in fewer cases, cerebral palsy [3]. Spastic tetraparesis, one of the most severe forms of cerebral palsy, is characterized by spasticity, dystonia, and sensorimotor integration deficits [5,6,7], particularly proprioceptive feedback, which further complicate the execution of coordinated and precise movements [8,9]. These impairments severely limit motor control, daily activities, and quality of life, emphasizing the need for advanced therapeutic approaches and multidisciplinary pharmacological (i.e., neurotoxins) and non-pharmacological (i.e., neuromodulation and rehabilitation) and highly integrated treatments to address the different symptoms and the interference of each one with motor control [10]. Six key elements were identified in the rehabilitation for this condition: (i) movement practice, (ii) training with constraint, (iii) sensory reorganization, (iv) normalization of muscle activity with external techniques, (v) neuromodulation with training, and (vi) compensatory strategies [11].

Emerging technologies, such as robotic systems and wearable neuro-suits, address these challenges by facilitating neuromodulation, enhancing motor function, and fostering neuroplasticity. Robotic devices like end-effectors and biomechatronic systems provide haptic feedback and virtual reality environments where patients can perform game-based exercises with immediate feedback on their progress, enabling precise assessment and rehabilitation of motor skills [12,13]. The interactive nature of these exercises fosters a sense of accomplishment and engagement, encouraging patients to participate actively in their recovery. The design of these devices often mimics natural sensory feedback mechanisms, enabling a bioinspired interaction between user and machine that optimizes rehabilitation outcomes [14]. Meanwhile, wearable neuro-suits deliver tailored electrical stimulation to reduce spasticity and promote functional recovery [15]. These garments integrate multiple electrodes for programmed electrical stimulation, specifically customized for each patient. Peripheral muscular neuromodulation aims to modulate spasticity through mechanisms of reciprocal inhibition, reducing the excitability of spastic muscles and promoting neuroplasticity in cerebral and spinal circuits [16]. Inspired by natural neural pathways, this mechanism reflects how the nervous system dynamically regulates muscle activity, underscoring the biomimetic nature of the intervention [17]. This innovative, non-invasive technique enhances the effectiveness of rehabilitation, improving motor function and supporting the synaptic reorganization of sensory and motor cortices [18].

Moreover, robotic neurorehabilitation offers significant potential in treating this condition, allowing specific motor difficulties to be addressed through a highly personalized and technologically advanced rehabilitation program [19]. Equipped with motors that assist or resist movements as needed, these devices not only facilitate movement execution but also provide accurate assessments of performance (i.e., a range of motion (ROM) and strength), adapting in real time to muscle activity. This adaptability ensures patients always receive appropriate support and feedback.

This adaptability closely mirrors the natural ability of the human neuromuscular system to respond dynamically to varying physical demands [20]. The integration of bioinspired principles in these technologies opens avenues for further advancements in biomimetics, potentially revolutionizing sensoric systems, neuroprosthetics, and adaptive devices that emulate biological processes [4].

This case report investigates the combined use of advanced robotics combined with a biomechatronic device in a young adult with dystonic spastic tetraparesis to enhance motor function and quality of life. This integrated approach aims to improve upper limb function, fine motor coordination, and autonomy in daily activities. By examining the bioinspired aspects and potential applications of these technologies, this study contributes to a broader understanding of how biomimetic principles can enhance neurorehabilitation practices [21].

## 2. Materials and Methods

This observational study was conducted as a prospective observation of a single case, following the CARE guidelines for case report presentation [22].

### 2.1. Case Description

**Figure 1:**
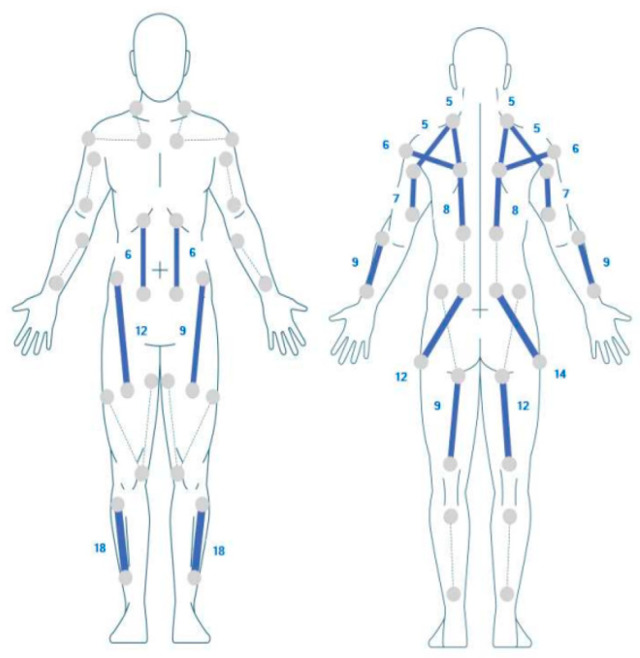
Neuro-suit stimulation map. The numbers are in microsecond scale (pulse width): “1” represents 24 microseconds, and for each subsequent unit, 5 microseconds are added. Blue lines indicate stimulation “on”, while gray lines indicate stimulation “off”.

The patient, a 31-year-old male with dystonic spastic tetraparesis due to neonatal trauma, exhibited notable limitations in activities of daily living (ADLs) and required assistance for ambulation and fine motor tasks. Pre-experiment assessments revealed dysarthric speech, sensory deficits mitigated by auditory and visual aids, and significant spasticity, particularly in the right upper limb [23]. Despite these challenges, he demonstrated partial independence in ADLs, reflecting the potential for targeted rehabilitation to improve functionality. Following an episode of physical and psychological overload, the patient experienced a significant decline in motor autonomy. Subsequently, he resumed an intensive physiotherapy program, supplemented by a neuro-suit (Exopulse Mollii Suit, Ottobock SE & Co., Duderstadt, Germany). The stimulation provided by the neuro-suit uses square wave stimulation at 20 V with a frequency of 20 Hz (Figure 1). This study was conducted in accordance with the ethical principles outlined in the Declaration of Helsinki. The patient provided written informed consent to participate in this study and for the use of the data for scientific purposes.

Rehabilitation program and integration of advanced robotic and biomechatronic devices.

The treatment program began in November 2022, with the regular use of the neuro-suit for a one-hour session, three times a week to reduce spasticity and enhance motor function. In December 2022, the neuro-suit treatment was supplemented with ten tailored sessions of robotic rehabilitation, designed to address the patient’s specific needs. This comprehensive program combining the neuro-suit with the Motore and Ultra+ robotic devices provided a synergistic approach to neurorehabilitation, gradually increasing complexity and difficulty aligned with motor learning principles, as explained above.

**Figure 2:**
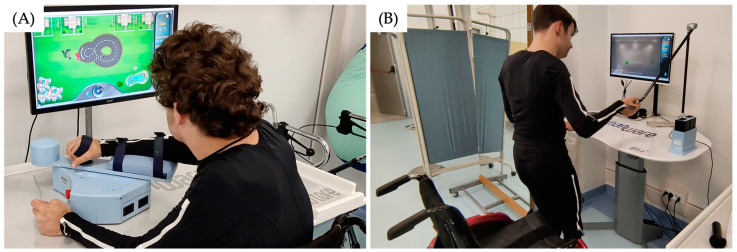
Patient interaction with the Motore (**A**) and Ultra+ (**B**) devices during rehabilitation tasks.

The Motore device (Humanware Srl, Pisa, Italy) is a robotic system characterized by a handgrip, integrated sensors for movement tracking, and a customizable resistance (Figure 2A). It supports upper limb motor rehabilitation by providing real-time visual and auditory feedback through a control unit and a graphical interface, enabling therapists to implement individualized neuromotor training programs. The Motore device operates in a 2D plane and includes forearm support, offering task-specific therapy in a compact format that can be adapted to various rehabilitation settings [24].

The Ultra+ biomechatronic device (Humanware Srl, Pisa, Italy) extends the capabilities of the Motore, offering additional features for upper limb training (Figure 2B). It comprises an articulated series of links with seven degrees of freedom, a handgrip incorporating force sensors, and a software designed for real-time motion analysis and therapy customization. Unlike the Motore, the Ultra+ provides functionality in both 2D and 3D environments [25].

This design facilitates more precise movement guidance and complex task-specific exercises, supporting activities for motor recovery, functional training, and cognitive enhancement. With greater precision, enhanced software features, and broader customization options, the Ultra+ is particularly suited for patients requiring intensive and advanced therapeutic interventions. Moreover, this device played a pivotal role in gradually transitioning the patient to more challenging therapeutic exercises (e.g., from the sitting to the standing position).

**Figure 3:**
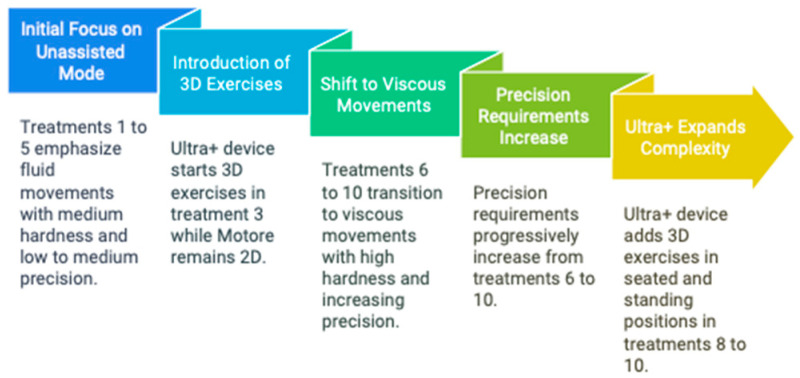
Rehabilitative treatment plan.

The rehabilitation program evolved progressively across the ten sessions, and it was tailored to the patient’s capabilities and progressively increased in complexity and difficulty aligned with motor learning principles. Initially, treatments 1 to 5 emphasized fluid movements in unassisted mode with moderate resistance and precision. As outlined in Figure 3, after these first five sessions, the treatment was shifted to viscous movements with increased resistance and precision demands by intensifying three key areas: (a) resistance provided by the devices, (b) the level of precision required, and (c) exercises performed in either 2D or 3D, in both sitting and standing positions. In particular, the Ultra+ device introduced 3D exercises starting in treatment 3, expanding to seated and standing positions by treatments 8 to 10, enhancing feedforward postural control related to upper limb function. These adjustments were meticulously planned to align with motor learning principles, ensuring that the patient’s rehabilitation was both challenging and achievable, promoting consistent progress toward functional recovery.

### 2.2. Clinical and Instrumental Assessments

To ensure objectivity, all evaluations were video-recorded and subsequently analyzed by the same operator, who was blinded to treatment details. The patient underwent assessments at four intervals: one month before initiating robotic treatment (T-1), immediately before rehabilitation (T0), at the end of the training program (T1), and one month post-rehabilitation (T2).

The Fugl-Meyer Assessment evaluated upper extremity motor recovery, with scores ranging from 0 to 66, where higher scores indicated better performance [26]. Trunk function and stability were measured using the Trunk Impairment Scale (TIS), which provides scores from 0 to 23, with higher scores reflecting improved performance [27]. Functional independence in daily activities was assessed using the Barthel Index (BI), which ranges from 0 (total dependence) to 100 (complete independence) [28,29,30]. Quality of life was evaluated with the Short Form Health Survey 36 (SF-36) questionnaire, encompassing various aspects of well-being, with scores ranging from 0 to 100, where higher scores indicate better quality of life [31]. At T1, the Clinical Global Impression (CGI) scale was administered to capture the patient’s subjective perception of progress [32] compared to the patient’s condition at admission (1: very much improved since the initiation of treatment; 2: much improved; 3: minimally improved; 4: no change from baseline; 5: minimally worse; 6: much worse; 7: very much worse).

Objective data were gathered from the biomechatronic and robotic devices utilized during the training sessions. Metrics for joint range of motion (ROM) include precision at work (percentage), distance reached (percentage), and playing time (seconds), while muscle strength metrics include the percentage of force achieved relative to the requirement and the duration of playing time. This comprehensive integration of clinical and instrumental metrics provided a robust framework for evaluating the patient’s rehabilitation progress.

## 3. Results

The study protocol proved feasible, with no discomfort-related issues (e.g., pain or fatigue) or other complications detected throughout the study period. All rehabilitation sessions and assessments were completed.

The clinical outcomes showed improvements across multiple domains. In the right upper limb, the Fugl-Meyer motor function score increased from 32 at T-1 and T0 to 46 at T1, slightly reducing to 41 at T2. Sensory and passive joint motion scores remained stable or slightly improved. In the left upper limb, motor function scores rose from 51 to 58 by T2, reflecting progress despite already high baseline scores. Functional independence, measured by the BI, increased from 60 at T0 to 85 by T1, maintaining this level at T2. The SF-36 results indicated improvements in several domains over time. Physical functioning increased from 50 at T-1 and T0 to 65 at T2, while role limitations due to emotional problems rose from 0 to 67. Emotional well-being improved from 68 to 80, pain increased from 90 to 100, and general health advanced from 75 to 85. These changes highlight positive trends in physical and emotional health outcomes. The TIS also displayed substantial progress, rising from 6 to 18 during the intervention. The CGI scale indicated a moderate improvement in the patient’s perception of their functional capabilities. Table 1 displays clinical assessment results.

**Figure 4:**
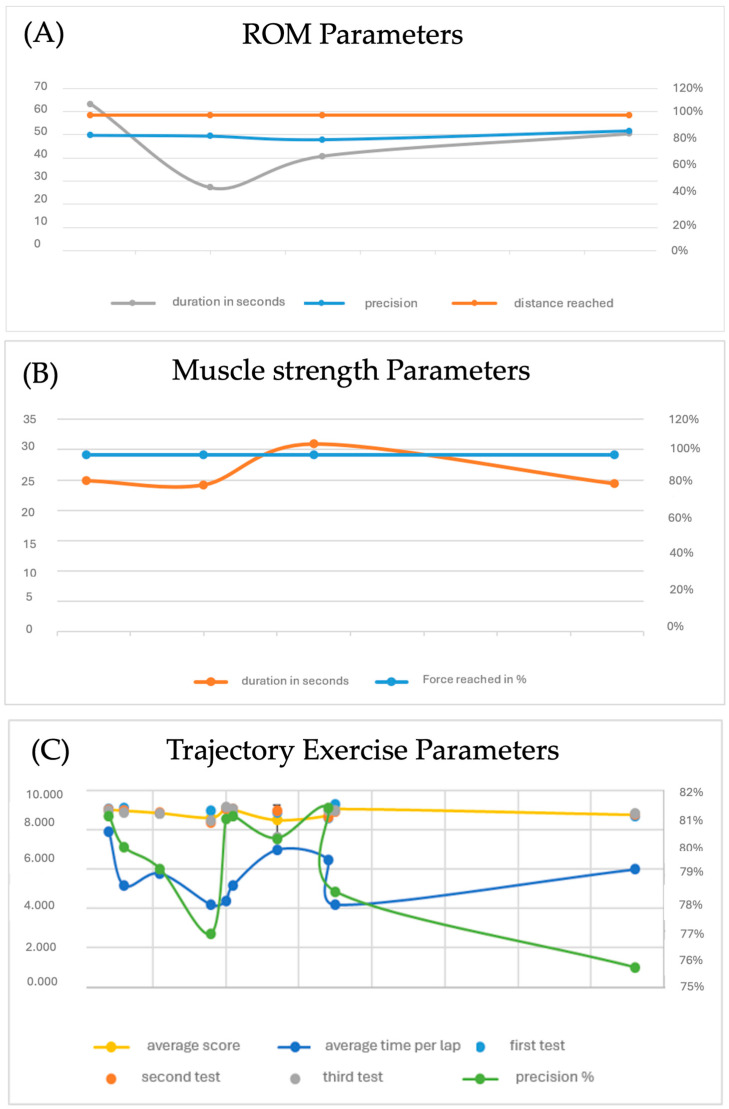
Evolution of performance metrics for ROM, muscle strength, and trajectory exercises across sessions. Legend: Panel (**A**) illustrates the changes in joint range of motion (ROM) performance metrics over time. Panel (**B**) displays the metrics for muscle strength tasks across sessions. Panel (**C**) represents the results of trajectory-based exercises. The *Y*-axis on the left represents values in seconds, while the *Y*-axis on the right shows percentage values related to the achievement of specific tasks. The *X*-axis represents the timeline across sessions.

Objective data from the biomechatronic and robotic devices further corroborated a stable performance in distance and force metrics, with minor fluctuations observed in precision and playing time over the course of the sessions despite increasing the task complexity (Table 2). The evolution of performance metrics measured by Motore is reported in Figure 4.

As shown in Figure 4, Panel A about ROM measurements revealed increased joint mobility during the training period, while metrics such as exercise scores, target accuracy, execution duration, and work precision showcased the patient’s ability to manage progressively challenging tasks. Panel B shows values related to muscle strength, which remained virtually constant throughout the study, indicating the maintenance of good performance. These findings highlight a stable performance in distance and force metrics, with minor fluctuations observed in precision and playing time over the course of the sessions despite increasing the task complexity. Panel C shows that, with the increasing difficulty of the exercises, a decrease in accuracy percentage was observed in some cases due to the need to restrict upper limb movement. However, scores related to tracking and trajectory exercises, as illustrated, remained stable, suggesting that the patient could adapt to new challenges with a stable success margin. This adaptation suggests ongoing neuroplasticity, a crucial aspect of long-term recovery.

## 4. Discussion

The study presented has suggested for the first time the effectiveness of combining robotic and biomechatronic devices and a neuro-suit in the rehabilitation process of a young adult with spastic dystonic tetraparesis in the clinical (non-experimental/lab) context. The results obtained through the Fugl-Meyer Scale demonstrated functional improvement and progress that surpasses the Minimal Clinically Important Difference (MCID) for chronic patients. This result is significant, as it highlights the effectiveness of the intervention in a clinical context where improvements are often slow and difficult to achieve. Integrating the neuro-suit and robotic and biomechatronic devices has optimized motor recovery by facilitating intensive repetition of specific exercises and continuous patient progress monitoring. Additionally, the TIS analysis revealed a significant improvement in trunk support function and body function projection. These advancements were observed through progressively more complex exercises, leading to better postural control and stability during functional activities (Figure 3). The patient demonstrated improved maintenance of an upright posture and dynamic balance—key aspects for autonomy in daily activities.

The BI and SF-36 scores indicated improved patient quality of life, with increased autonomy in daily activities and enhanced participation in bimanual tasks. These improvements were accompanied by an increase in the patient’s confidence in their abilities, an important psychological factor in the rehabilitation context. The perceived greater autonomy positively impacted not only the physical aspect but also the patient’s emotional well-being, fostering greater motivation and adherence to the rehabilitation program. Evaluations of ROM and muscle strength, as reported in Figure 4, showed consistent results, suggesting that the patient maintained good performance even during the more challenging phases of treatment. This consistency in results attests to the patient’s ability to adapt to increasing levels of exercise difficulty, indicating improved muscle strength and endurance and progressive motor control. With the increasing difficulty of the exercises, a decrease in accuracy percentage was observed in some cases due to the need to restrict upper limb movement. However, scores related to tracking and trajectory exercises, as illustrated in Figure 4, remained stable, suggesting that the patient could adapt to new challenges with a stable success margin. This adaptation might be supported by ongoing neuroplasticity, a crucial aspect of long-term recovery.

This study highlights the potential application of combining robotic and biomechatronic devices in rehabilitation, particularly by leveraging bioinspired principles. These technologies can mimic natural and musculoskeletal dynamics and have significant implications for the development of biomimetic systems. Robotic and wearable technologies represent a significant frontier in neurorehabilitation, offering innovative tools to improve the quality of life for patients with motor disabilities [33]. By integrating adaptive control mechanisms and real-time feedback, the intervention might emulate biological processes enabling precise and personalized rehabilitation tailored to individual needs. This bioinspired adaptability aligns with the capability of the human nervous system to respond dynamically to varying demands, paving the way for advancement in sensorics systems, periprosthetics, and adaptive devices. However, the practical integration of these systems is not without challenges [33]. One of the main obstacles lies in the synchronization between various devices, which can affect the fluidity and naturalness of movements during therapy sessions. Added to this is the complexity of the configuration process: accurately calibrating the devices for each individual requires advanced technical skills and considerable time. Such requirements may limit the accessibility of these solutions, especially in clinical settings with limited resources. In terms of information processing systems, the possibility to have real-time monitoring and data analysis capabilities would offer valuable insights for developing intelligent algorithms in rehabilitation technologies. These algorithms could refine the interaction between the clinicians and the device, making the system more responsive with the principles of physiological motor learning. The integration of bioinspired feedback mechanisms into robotics systems is promising for advancing the field of biomimetics into rehabilitation.

Finally, feedback from the patients and therapists highlights the importance of integrating advanced technologies into rehabilitation, emphasizing both the observed improvements and the practical challenges associated with using robotic devices. The findings of this study can, therefore, inform the design of future bioinspired technologies. For instance, the haptic feedback and task-oriented exercises provided by the robotic devices can inspire innovations in sensory processing systems, enabling devices to interact more automatically with users. In addition, the wearable neuro-suits tailored stimulation approach illustrates the potential for neuromodulation technologies that mimic natural neural pathways, facilitating neuroplasticity and functional recovery.

This study’s main limitation is its observational nature, which limits the generalizability of its findings. However, observational studies are essential for generating research hypotheses before undertaking time- and cost-intensive interventional studies. Notable strengths include a baseline assessment, a comprehensive assessment battery that explores effects within the ICF framework, and the blinding of evaluations. Despite the evident benefits, the patient encountered frustrations related to the mechanical limitations of robotic and biomechatronic devices. These issues often stemmed from a lack of perfect synchronization between the devices and the patient’s spontaneous movements, which sometimes hindered the fluidity of gestures and created a sense of restriction. This mismatch highlighted a critical area for improvement in the future development of these technologies. Enhancing the responsiveness and adaptability of robotic and biomechatronic devices to better align with human movement’s complex and dynamic nature will be essential in maximizing their therapeutic potential. Moreover, the practical challenges encountered during therapy, such as the considerable amount of time required to use and adjust the neuro-suit and the management of technical issues, cannot be overlooked. While secondary to the primary goal of functional recovery, these aspects are significant in terms of the overall user experience and the feasibility of widespread adoption of these technologies in clinical settings. Addressing these challenges will be crucial for making these interventions more accessible and user friendly. The reported improvements in both general motor abilities and fine coordination, particularly in the previously impaired right arm, underscore the effectiveness of this approach. The ability to perform movements with greater precision and control contributed to measurable physical progress and had a profound psychological impact, providing the patient with a renewed sense of autonomy and hope for recovery.

## 5. Conclusions

The integration of advanced technologies, including the neuro-suit and robotic and biomechatronic devices, has demonstrated potential in enhancing motor function and quality of life in the management of spastic dystonic tetraparesis. This case emphasizes the feasibility of creating a unified framework for rehabilitation, including robotics, wearable technologies, and adaptive sensors, underscoring the broader applicability of bioinspired technologies in neurorehabilitation. Despite the challenges faced, including technical and logistical barriers, the positive results emphasize the importance of continued exploration and refinement of robotic, biomechatronic, and wearable technologies to optimize their adaptability and integration into therapeutic practices. Future research should prioritize improving usability, individual customization, and seamless integration into clinical workflows. With ongoing technological advancements, these solutions have the potential not only to enhance physical recovery but also to significantly improve the overall quality of life for individuals facing complex motor impairments. Ultimately, this approach represents a promising step forward in creating comprehensive, patient-centered rehabilitation strategies. Future advancements in bioinspired technologies could expand their application beyond spastic tetraparesis, potentially offering personalized solutions for a variety of motor disorders and fostering greater adaptability in therapeutic devices. The biomimetic principles underlying these technologies may also contribute to the development of more sophisticated neuroprosthetic and sensoric systems, allowing for dynamic, patient-specific interventions that emulate natural biological processes.

## Figures and Tables

**Table 1:** Longitudinal assessment of motor function, independence, and quality of life through clinical outcomes.

		T-1	T0	T1	T2
Fugl-Meyer Upper Limb	Right				
	Motor function (0–66)	32	32	46	41
	Sensation (0–12)	12	12	12	12
	Passive joint motion (0–24)	19	19	23	20
	Joint pain (0–24)				
	Left				
	Motor function (0–66)	51	51	52	58
	Sensation (0–12)	12	12	12	12
	Passive joint motion (0–24)	24	24	24	24
	Joint pain (0–24)	23	23	21	22
TIS (0–23)		6	6	18	18
BI (0–100)		60	65	85	85
SF-36 (0–100)					
	Physical functioning	50	50		65
	Role limitations due to physical health	100	100		100
	Role limitations due to emotional problems:	0	0		67
	Energy/fatigue	75	75		60
	Emotional well-being	68	68		80
	Social functioning	75	75		75
	Pain	90	90		100
	General health	75	75		85
CGI (0–7)				2	2

Legend: TIS, Trunk Impairment scale; BI, Barthel Index; SF-36: Short Form Health Survey 36 Questionnaire; CGI, Clinical Global Impression; T-1, one month before initiating robotic treatment; T0, immediately before rehabilitation: T1, at the end of the training program; T2, one month post-rehabilitation. The range of scores is displayed in brackets.

**Table 2:** Performance metrics for joint range of motion and muscle strength across multiple sessions.

	First Session	Fifth Session	Last Session	Follow-Up
Joint ROM				
Precision at work (%)	85.1	84.1	81.9	88.4
Distance reached	100	100	100	100
Playing time (s)	63.1	27.1	40.7	50.3
Muscle strength				
Force achieved/required (%)	100	100	100	100
Playing time (s)	24.9	24.2	30.9	24.4

Legend: ROM, range of motion; %, percentage; s, seconds. The last session and follow-up correspond to T1 and T2, respectively.

## Data Availability

Dataset available on request from the authors.
